# Molecular and Cellular Mechanisms of Sperm-Oocyte Interactions Opinions Relative to *in Vitro* Fertilization (IVF)

**DOI:** 10.3390/ijms150712972

**Published:** 2014-07-22

**Authors:** George Anifandis, Christina Messini, Konstantinos Dafopoulos, Sotiris Sotiriou, Ioannis Messinis

**Affiliations:** Department of Obstetrics and Gynaecology, School of Health Sciences, Faculty of Medicine Larisa, University of Thessaly, Larissa 41110, Greece; E-Mails: pireaschristina@gmail.com (C.M.); kdafop@yahoo.com (K.D.); sotirious@med.uth.gr (S.S.); messinis@med.uth.gr (I.M.)

**Keywords:** oocyte, spermatozoa, fertilization, *in vitro* fertilization (IVF)

## Abstract

One of the biggest prerequisites for pregnancy is the fertilization step, where a human haploid spermatozoon interacts and penetrates one haploid oocyte in order to produce the diploid zygote. Although fertilization is defined by the presence of two pronuclei and the extraction of the second polar body the process itself requires preparation of both gametes for fertilization to take place at a specific time. These preparations include a number of consecutive biochemical and molecular events with the help of specific molecules and with the consequential interaction between the two gametes. These events take place at three different levels and in a precise order, where the moving spermatozoon penetrates (a) the outer vestments of the oocyte, known as the cumulus cell layer; (b) the zona pellucida (ZP); where exocytosis of the acrosome contents take place and (c) direct interaction of the spermatozoon with the plasma membrane of the oocyte, which involves a firm adhesion of the head of the spermatozoon with the oocyte plasma membrane that culminates with the fusion of both sperm and oocyte membranes (Part I). After the above interactions, a cascade of molecular signal transductions is initiated which results in oocyte activation. Soon after the entry of the first spermatozoon into the oocyte and oocyte activation, the oocyte’s coat (the ZP) and the oocyte’s plasma membrane seem to change quickly in order to initiate a fast block to a second spermatozoon (Part II). Sometimes, two spermatozoa fuse with one oocyte, an incidence of 1%–2%, resulting in polyploid fetuses that account for up to 10%–20% of spontaneously aborted human conceptuses. The present review aims to focus on the first part of the human sperm and oocyte interactions, emphasizing the latest molecular and cellular mechanisms controlling this process.

## 1. Sperm Migration into the Human Female Oviduct

**Figure 1:**
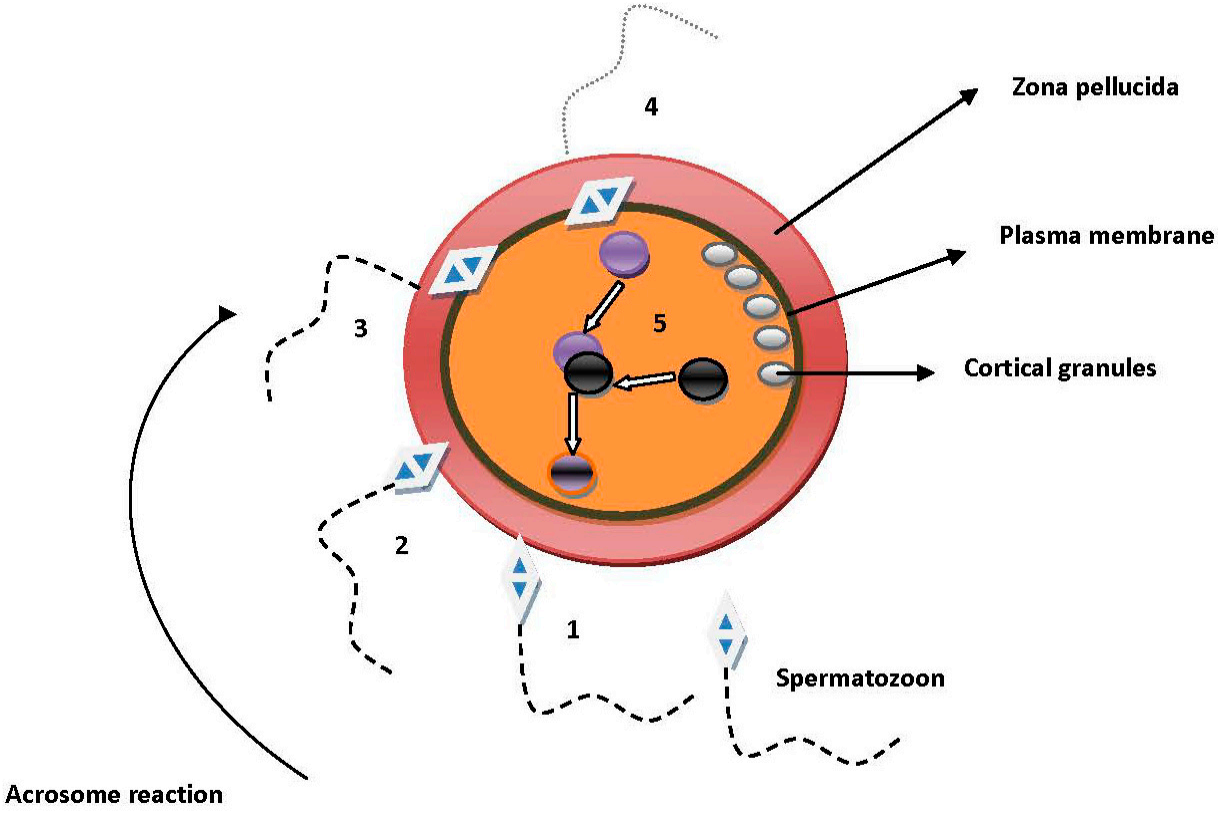
The fertilization process. (**1**) The spermatozoon is attached and bound to the zona pellucida (ZP) after the successful penetration of the cumulus matrix. It is believed that the cumulus mass secrets chemoattractants for the spermatozoa to “locate” the ovulated oocyte. Soon after “locating” the oocyte, the acrosome-intact spermatozoa begin the acrosome reaction process. Molecules of the acrosomal matrix, such as zonadhesin and sp56, have been proposed to play significant roles in both the binding and adhesion processes. Acrosome-intact spermatozoa adhered and bound to an epitope of the ZP3 and especially to ZP3 O-linked oligosaccharides, which has been characterized as sperm combining site; (**2**) The penetration of the ZP. It has been suggested that penetration of the ZP can be achieved only through acrosome-reacted spermatozoa. With the exception of the ZP3 protein of the oocytes, acrosin and testicular serine protease 5 (TESP5) are the candidate factors (from the sperm side) that are found to participate in the process of ZP penetration. We should bear in mind that spermatozoa that bind to the ZP are not acrosome-reacted and not all acrosome-reacted spermatozoa penetrate the ZP. This may imply that there are maybe different populations in the same semen sample that can explain the above situation; (**3**) Adhesion of sperm with oocyte plasma membrane. The site of the spermatozoon that binds and subsequently fuses with the plasma membrane of the oocyte is the equatorial segment. From the oocyte side, cluster differentiation 9 (CD9) and α6β1 integrin have been shown to play a major role in the adhesion process. From the spermatozoon side, cysteine-rich secretory protein 1, known as cysteine-rich secretory protein 1 (CRISP1) has been recognized as the most potential molecule participating in the adhesion procedure. The exact mechanism seems to be related with the matching (pairing) of surface molecules from both sides; (**4**) Fusion process. Following the adhesion the process is finalized by the formation of a fusing pore (at the site where microvilli are enough to support the fusion process) and the cytoplasmatic continuity of both gametes. IZUMO1 has been proposed to contribute significantly to the fusion process. Although it has been established that this molecule does not include any fusogenic domain, it seems that in combination with other equatorial segment proteins, known as SPESPs (sperm equatorial segment proteins), it participates in the fusion process. Lately, it has been discovered to be the IZUMO1 partner, Juno. The interaction of IZUMO1-Juno seems to be essential for the adhesion process, promoting in this way the fusion procedure. Although it is obvious that fertilization is a multi-complex process it has not yet been elucidated whether each step is dependable upon the previous step. In other words, once one potent fertilizing spermatozoon binds to the ZP will it end with the fusion process? Because all experimental studies concern thousands or millions of spermatozoa it not clear whether the above question can be resolved, but it is likely that due to the multi-step character of the fertilization process each step depends very much on the previous step; (**5**) Male and female pronuclei are coming together forming the nucleus of the zygote. Soon after the fusion process a signal transduction cascade is initiating (involving Ca^2+^) and results in altering the ZP and the plasma membrane in order to block the polyspermy situation. Cortical granules (located beneath the plasma membrane of the oocyte) seem to have the basic role in this final step. It is well known that exocytosis of cortical granules contributes to the above process.

In order for fertilization to occur, human sperm should migrate a relative long distance to reach the fertilization point through the female reproductive tract. It is obvious that during this trip the spermatozoa should overcome a variety of barriers, beginning with the navigation to the three segments of the oviduct, uterotubal junction (UTJ), isthmus and the ampulla, and upon arrival to the fertilization point, end up with the penetration of the oocyte’s extracellular vestments, consisting of the cumulus cells, the zona pellucida (ZP) and oocyte’s plasma membrane (Figure 1). At the beginning there was the impression that the migration process was due to the ability of the spermatozoa to move, but several lines of evidence gave another explanation, that migration of the spermatozoa through the segments of the oviduct is molecularly regulated. Most of the factors involved have been studied extensively in knockout mice. It is known for example that spermatozoa of male mice that lack one of the regulatory molecules, calmegin (clgn), sperm a disintegrin and metalloproteinases (ADAMs, including fertilin α (ADAM1), fertilin β (ADAM2) and cyritestin (ADAM3)) and the angiotensin-converting enzyme (ACE), maintain their ability to move but are unable to migrate through the oviduct [1,2,3,4,5].

Calmegin, is a testis-specific chaperone, which is located in the endoplasmatic reticulum of the testis cells and interestingly disappears gradually during spermatogenesis [6]. This means that after ejaculation the sperm surface is free of the molecule calmegin, implying that the specific molecule does not play any direct role in the migration process, but it is possible that it promotes the expression of other proteins that finally interact with the oviduct epithelium. The molecule defensin β 126 (DEFB126), a cysteine-rich cationic polypeptide, has been suggested to promote penetration of the cervical mucus due to its overall negative charge [7], but until now its role as well as the role of other defensins remains to be clarified. Furthermore, fibronectin type II proteins, known as binders of sperm surface proteins (BSPs), such as binding sperm surface protein H1 (BSPH1) [8], seem to play a major role in sperm storage in the isthmus, since it has been found that they interact with annexin family proteins of the oviduct epithelial surface [9,10]. Oviduct migration is delayed in the isthmic epithelium, where the isthmic environment stabilizes the spermatozoa for a relatively small period of time [11]. Destabilization of spermatozoa from the isthmic epithelium has been proposed to occur due to flagellum movements, or to sperm molecules that lose their ability to attach to the oviduct epithelium [12,13]. For the latter proposal, catsper1 deficient spermatozoa are incapable to detach from the oviduct epithelium by controlling the influx of Ca^2+^ concentrations, necessary for the hyperactivated state, which causes detachment from the epithelium. Besides, mutations in the *catsper1* gene have been suggested to play a direct role in human male infertility [14,15]. Leaving the isthmus epithelium, spermatozoa are competent to inseminate the oocytes (capacitation process) and subsequently move to the ampulla in order for fertilization to occur. Collectively the above reports indirectly support the notion that the isthmus epithelium reduces the number of spermatozoa that will arrive at the fertilization point and secondly reduces the chances for polyspermy. These proposals are not very possible because the number of spermatozoa that arrive at the fertilization point is depending very much upon the spermatozoa that are capable to fertilize (capacitated), while in a semen sample it is impossible for all the spermatozoa to gain this ability. From the *in vitro* fertilization (IVF) point of view, during semen preparation for IVF/Intracytoplasmatic Sperm Injection (ICSI) treatments only a portion of the initial semen sample will be able to fertilize. Probably, the detachment from the isthmus epithelium is associated with the capacitation process. Secondly, the chances for polyspermy are depending very much upon the mechanisms the oocyte develops in order to block the polyspermy situation and not upon the number of spermatozoa. This notion is derived from our experience in IVF insemination, where the number of ready for acrosome reaction spermatozoa (80,000–100,000 sperm cells/ Cumulus Oocyte Complex (COC)) almost never develops the polyspermy phenomenon, while whenever it is happening, it is attributed to ZP deficiency.

**Figure 2:**
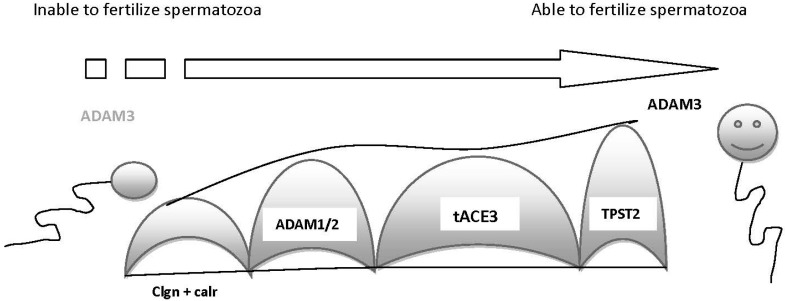
Schematic presentation (tunnel) of the obstacles the spermatozoon should overcome in order for ADAM3 to be expressed. The expression of ADAM3 is essential for spermatozoon to transition from inability to ability to fertilize. In order for ADAM3 to be expressed, several different genes have been implicated. The disruption or the partial function or the dysfunction of one of these genes results in a range of infertility, beginning with sub-fertility and ending with total infertility. Among the molecules that contribute to the expression of ADAM3, calmegin (clgn) and calsperin (calr) (testis-specific chaperones) are required. Moreover, the formation of a heterodimer of ADAM1 and ADAM2 is significant and this formation depends on the function of clgn. Last, tpst2 has been implicated in the expression of ADAM3, while t-ace3 has been found to play role in the final distribution of ADAM3 on the sperm surface.

Fertilin α, fertilin β and cyritestin are proteins that have significant homologies and conserved structures as so far known members of a disintegrin and metalloproteinases (ADAM) family, being ADAM1, ADAM2 and ADAM3, respectively. Their homology is based upon their specific disintegrin and metalloprotease domains. All the above ADAM proteins have been located in regions of the sperm head [16,17,18]. After the conduction of gene experiments it was found that ADAM1 may be expressed as 2 type, ADAM1a and ADAM1b [19], while ADAM1b and ADAM2 form a heterodimer in the sperm surface [20,21]. Although the above heterodimer has been reported to function in the general process of sperm–oocyte interaction [22], thus indicating this heterodimer as an essential molecule for fertilization, later it was discovered that spermatozoa with deficiency in the *ADAM2* gene exhibited extreme difficulty to migrate through the oviduct [21]. Although these spermatozoa exhibit normal motility (in terms of movement) and physiological fertilization, it can be easily concluded that these spermatozoa are incapable to migrate or to interact with the oviduct epithelium. In contrast, ADAM3 knockout spermatozoa showed physiological migration through the oviduct [23], but a recent report indicated no sperm migration through the oviduct in ADAM3-null spermatozoa [5]. All the relative gene experiments with knockout spermatozoa resulted in dramatic impairment of the ADAM3 function. It seems that ADAM3 plays a major, if not key role, in the function of transit through the oviduct and ADAM’s expression depends upon the concerted function of a group of other proteins, some of them addressed above. Moreover, abnormal expression of these proteins seems to result in abnormal expression of ADAM3 on the sperm surface, which in terns result in impairment of oviductal migration (Figure 2). The molecule ACE is one of the proteins that contribute to the expression of ADAM3. Studies with ACE knockout spermatozoa have shown similar defects in terms of migration into oviduct and this phenotype was explained by the absence of ADAM3 in the sperm surface [24].

Conclusively, regarding the molecules that contribute to the sperm migration through the oviduct, it can be postulated that ADAM3 is the most important factor that plays a key role in sperm migration. Disruption of several different genes (some mentioned above and some others in Figure 2) seems to produce spermatozoa unable to transit through the oviduct or incapable to attach to oviductal epithelium. ADAM3 expression on the sperm surface requires the co-operation/co-expression of the above genes. Although the exact mechanism has not been yet clarified, it is possible that the expression of each gene is essential for the expression of the next gene, promoting in this way the normal expression of the ADAM3 protein. Complete or partial deficiency of one of these genes seems to result in a complete or partial infertile phenotype.

## 2. Molecules Involved in Sperm–Oocyte Interactions

### 2.1. Sperm and Cumulus Mass Interactions

After the successful detachment from the ishmus, the spermatozoa are moving to the last segment of the oviduct, the ampulla, where the spermatozoa seem to “identify” the presence of the oocyte by “locating” the COCs. This “identification” seems to act in a chemoattractant manner, since it has been reported that follicular fluid factors and COCs include and secrete, respectively, chemoattractants [25,26], promoting in this way the capacitation process. Moreover, olfactory family 1 proteins that are present as receptors on the COCs have been suggested to act as chemoattractants [27], implying that sperm, through some molecules, “smell” the molecules presented or secreted by the COCs.

**Figure 3:**
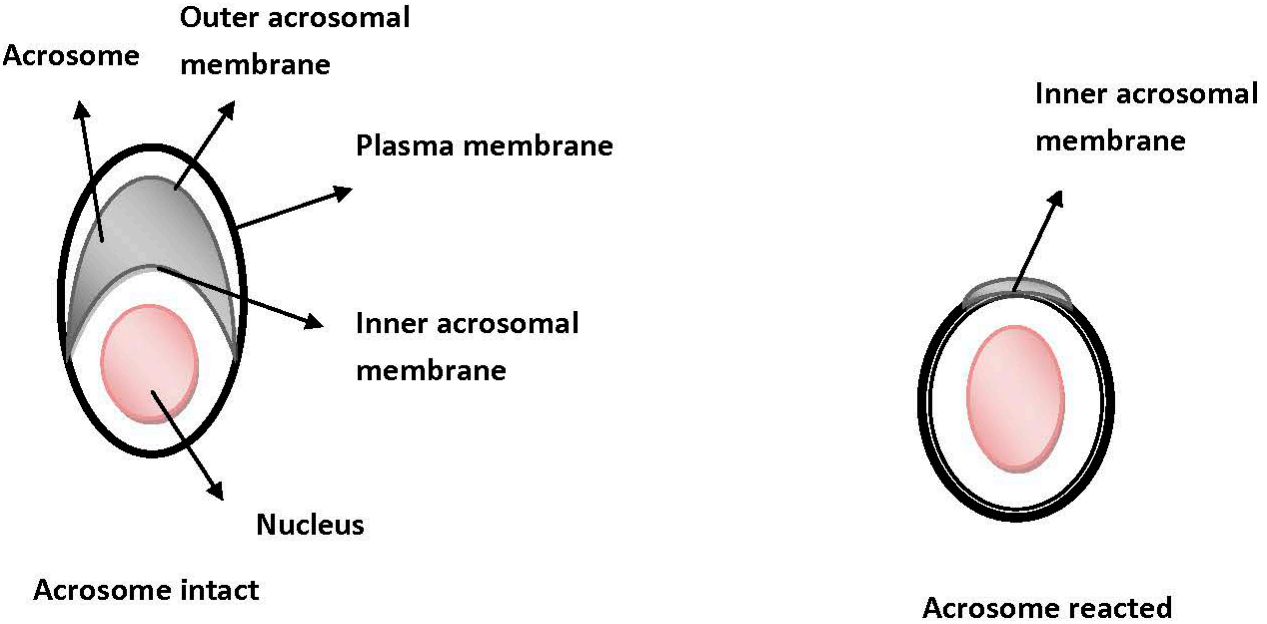
The process where an acrosome intact spermatozoon becomes acrosome-reacted. It is believed that the acrosome intact spermatozoa reach the ZP. The latter and especially *ZP3* has been proposed to promote the acrosome reaction. Following binding, the outer acrosomal membrane fuses with the sperm plasma membrane for the acrosomal contents (enzymes, such as acrosin) to be released for penetration of the ZP.

Human ovulated oocytes are surrounded by two distinct layers of cells, the outer layer of cumulus cells and the ZP. Beneath these two substantial layers the spermatozoon should reach and interact also with the plasma membrane of the oocyte in order for the fusion process to take place. COCs have been proposed to have a beneficial role in the fertilization procedure, as it has been found that disruption of genes involving the synthesizing or stabilization of COCs, result in a phenotype with reduced fertilization performance [28,29]. The cumulus matrix has been reported to promote sperm acrosome reaction [30], since the cells of cumulus oophorus are embedded in an extracellular matrix which consists mainly of proteins and carbohydrates, among which hyaluronan is the most known as a no-sulfated glycosaminoglycan. Sperm acrosome reaction, part of the process that is known as capacitation, has been characterized quite well. Briefly, acrosome reaction is an exocytosis procedure and occurs only in capacitated spermatozoa. Soon after ejaculation, capacitation takes place and is characterized by the removal of cholesterol and other sterols from the sperm surface. Following capacitation, the acrosome releases enzymes, among of which hyaluronidase seems to be the most responsible for the degradation/hydrolation of the hyaluronan of the cumulus oophorous. Hyalouronidase has been found to exist in two isoforms in epididymal spermatozoa, the Ph-20, a glycosylphosphatidylinositol (GPI)-anchored protein and Hyal5 [31,32,33]. Ph-20 is present on the plasma membrane of acrosome-intact spermatozoa, while Hyal5 is located on both acrosomal and plasma sperm membrane. Both molecules appear to play an important role in the degradation of the cumulus matrix. Specifically, Ph-20 is initially located on the plasma membrane of acrosome-intact spermatozoa, and as the acrosome reaction proceeds, it moves onto the inner acrosomal membrane of the acrosome-reacted spermatozoa [34] (Figure 3). This migration of Ph-20 during the acrosome reaction is thought to explain the protease activity of Ph-20 through the cumulus mass. Large amounts of Hyal5 are secreted during the acrosome reaction, thus implying that Hyal5 functions mostly for the hyaluronan hydrolysis of the cumulus mass. Spermatozoa of male mice in which Ph-20 was disrupted were still capable of fertilizing [32], suggesting that Ph-20 contributes most to the penetration of the cumulus matrix and barely to the late stages of fertilization. The reduced fertilization rate in Ph-20-deficient spermatozoa appears to be attributed to the difficulty of these spermatozoa to penetrate the cumulus oophorous. Last, given the co-operation of the two molecules, it has been suggested that Hyal5 disperse the cells from the cumulus matrix in order for Ph-20 to digest the cumulus cells [33]. Although sperm hyaluronidase has not been well characterized, and despite that sperm penetration through the cumulus cell layers is essential for fertilization, the exact molecular mechanisms that control the above steps needs to be clarified with gene-manipulated studies.

### 2.2. Sperm Binding, Adhesion and Penetration of the Zona Pellucida (ZP)

The second obstacle that spermatozoa should overcome is the ZP. Among the three major components of the ZP (*ZP1*, *ZP2* and *ZP3*), *ZP3* has been proposed to interact with sperm surface molecules. There are many reports that have investigated the role of *ZP3*. Laboratory experiments revealed that *ZP3* knockout female mice failed to have a ZP around their growing oocytes, thus resulting in infertility [35,36]. Also, it seems that the absence of the ZP impairs oocyte growth and follicle development, reducing in that way the number of fully-grown oocytes [37]. Heterozygous null female mice (*ZP3*^+/−^) are fertile but not at the same level as wild type female mice, because these mice maintain a thinner ZP (~2.7 μm, compared to ZP of wild type ~6.2 μm) [38]. Given that only capacitated spermatozoa bind to oocytes that have various ZP widths, it is obvious that *ZP3* is essential for the assembly of ZP, through the formation of heterodimer between *ZP1* and *ZP2*, independently of the thickness.

*ZP3* is composed of a polypeptide region of 424 amino acids (AA) to which are added a serine/threonine- (-*O*) and an asparagine- (-*N*) linked oligosaccharides [39]. *ZP3*, as also the other ZPs, has another region, which is called the ZP domain. There have been a variety of experimental studies to find out in which site of the *ZP3* the spermatozoon binds. This site was called the sperm combining-site and it is the epitope where a molecule(s) from the spermatozoon side matches with specific domains of *ZP3*. Investigation of this site revealed that acrosome-intact spermatozoa are adhered and bound to the *ZP3* O-linked oligosaccharides [40] and especially to a *ZP3* polypeptide that is encoded by exon-7 in the carboxy-terminal region and not to the polypeptide of the 424 AA [41]. Nevertheless, the polypeptide was essential for the completion of the acrosome reaction [42], because it has been proposed that *ZP3* induces the acrosome reaction and then the spermatozoon penetrates the ZP [30]. Furthermore, acrosome-reacted spermatozoa appeared to be able to penetrate the ZP [43], but it also well known that spermatozoa that bind to the ZP are not acrosome-reacted and not all acrosome-reacted spermatozoa penetrate the ZP, implying that the plasma membrane of acrosome-intact spermatozoa binds to the *ZP3*. The binding of spermatozoa to *ZP3* appears to activate a cascade of intracellular signals that culminate in the alteration of the intracellular Ca^2+^ concentrations and pH, something that is essential for acrosome exocytosis. In contrast to reports indicating that *ZP2* has no direct role in the binding process [41], there are reports suggesting that *ZP2* gains affinity to acrosome-reacted spermatozoa [44]. Collectively, there is no consensus regarding the exact mechanism of acrosome reaction by the ZP, but it is very possible that particular epitopes of the ZP (*ZP1*, *ZP2* and *ZP3*) initiate the acrosome reaction and the carboxy-terminal region of the *ZP3* is responsible for the completion of acrosomal exocytosis. Moreover, the acrosomal matrix consists of proteins that during or following capacitation are exposed to the sperm surface interacting with the ZP of the oocytes. Among these proteins, sp56 and zonadhesin [45,46,47] have been characterized.

Zonadhesin has gained much attention. Is a multiple domain protein that is produced during spermatogenesis and is located at the outer acrosomal membrane of spermatozoa [47,48], but as soon as an acrosome reaction takes place, zonadhesin disappears. Recent evidence indicates that the adhesion, between spermatozoa and oocytes, is significantly reduced when zonadhesin antibodies are present [49], implying that following capacitation, exposure of zonadhesin during acrosomal exocytosis is essential for ZP binding and adhesion. This last proposal supports the notion that the process of binding and adhesion is a matter of acrosome reaction, which initiates before binding to the ZP and is completely contrary to the dogma that ZP induces acrosomal exocytosis [50,51]. Further investigations concerning human zonadhesins identified 19 polymorphisms, which change the hydrophobicity of the molecule and subsequently affects its function [52]. Five different mRNAs variants of human zonadhesin have been isolated, and each of them seems to alter the capability of spermatozoa to bind/adhere to ZP. This information raises the possibility that spermatozoa carrying one at least of these variants may perform poorly in adhesion or even in fertilization. Given that not all spermatozoa of a semen sample are capable of fertilizing oocytes, the above proposal explains why so many spermatozoa are unable to reach the ovulated oocyte or result in an unsuccessful adhesion or penetration of the ZP. The result of this “pathological” situation may be idiopathic or unexplained infertility.

As far as sp56 is concerned, it was identified nearly 20 years ago and has been characterized as a sperm protein of the acrosomal matrix and as a member of the C3/C4 superfamily of binding proteins as a binding partner for the zona pellucida 3 receptor (ZP3R) of acrosome-intact spermatozoa. Recent evidence revealed that sp56 appears not be involved in sperm–zona pellucida binding or that this process might be functionally redundant [53]. sp56 is a component of the sperm's acrosomal matrix and is released to the sperm surface during capacitation. This explains why incapacitated spermatozoa are unable to adhere to the oocyte ZP [54], something which was further supported by other experimental studies underlying the essential role of ZP3R/sp56 in mediating the binding of sperm to the zona pellucida [55].

ZP penetration involves at least three different well-characterized sperm enzymes. The first one is acrosin, which is an acrosomal protease [56], the second one is GPI-anchored on the spermatozoon membrane serine protease, TESP [57] and the third is located in the acrosome and is a multi-subunit proteasome with proteolytic features [58]. It was first believed that acrosin was the only candidate factor that can cause the penetration of the ZP, but after the conduction of experimental studies it was found that protease activity remained in the acrosine (ACR) knockout spermatozoa [59]. The protease activity was attributed to a 42 kDa molecule and the attempt to identify this molecule resulted in five different serine proteases, TESPs (TESP1, TESP2, TESP3, TESP4 and TESP5), known as testicular serine proteases. Among these five proteases only TESP5 corresponds to the 42 kDa molecule and is the 21st member of the serine protease family PRSS21 [57]. Acrosine knockout spermatozoa displayed a delay in penetration of the ZP and this delay was attributed to the delay in dispersal of acrosomal components during acrosomal exocytosis [60]. Although spermatozoa lacking PRSS21 display normal fertility they also display severe defects concerning the penetration of the ZP. Both acrosin and PRSS21 knockout spermatozoa were not totally infertile (sub-fertile) but were unable to penetrate the ZP [61]. It is very possible that these two enzymes (ACR and PRSS21), with similarity in substrate specificity, may interact with each other during the penetration of the ZP [60]. This speculation seems to be reasonable since acrosine accelerates the activation of PRSS21 [62]. Uterine fluids seem to partially rescue the infertility status of acrosine and PRSS21 knockout spermatozoa, implying that a compensatory mechanism for the loss of both ACR and PRSS21 exists. It is very possible that PRSS21-like proteases or other unknown molecules may be secreted from the uterus or the oviduct into uterine fluids and compensate for the loss of ACR and PRSS21. Alternatively, a “mechano-sensory signal transduction” model [63] has been proposed which explains the continuation of sperm motility in order to bind on the ZP surface.

### 2.3. Sperm and Oocyte Plasma Membrane Interactions

After penetration of the ZP, the spermatozoon should overcome another obstacle, the oocyte plasma membrane. The spermatozoon plasma membrane fuses with the respective plasma membrane of the oocyte. It has been reported that only acrosome-reacted spermatozoa can fuse with the oocyte plasma membrane, implying that sperm molecules involved in membrane fusion are not yet active and become active upon exposure to the oocyte plasma membrane. It is very possible that acrosome reaction and penetration of the ZP triggers the initiation of cascade signals that promote the activation of the so far inactivated molecules that contribute to sperm fusion.

Cluster differentiation (CD) molecules are sperm and oocyte membrane surface clusters of differentiation molecules. Experimental studies demonstrated that some CD molecules are involved in the fusion process since these molecules are expressed in cells of the male and female genital track [64]. CD molecules are members of the tetraspanin superfamily, introduced as the “tetraspanin web” [65], because they form a multi-molecular “network”, a characteristic of all tetraspanins, by interacting with other proteins of the oocyte’s plasma membrane. The CD9 molecule contains four transmembrane domains that separate two extracellular domains of unequal size [66]. The first indication that CD9 is essential for the fusion procedure resulted from experimental studies using antibodies against CD9 [67], following a dose-dependent inhibition of sperm fusion. Given that the surface of the oocytes is covered by microvilli [68], CD9 is distributed all over the surface of the oocytes except the area lacking microvilli, which is the region overlying the mitotic spindle [69]. This information allows us to suggest that sperm–oocyte fusion is happening at the region where CD9 is abundantly expressed and not at the amicrovillar region, implying that microvilli may participate in the sperm–oocyte fusion process. Nevertheless, CD9 knockout oocytes have a different length, thickness and even density of their microvilli [70]. CD9 knockout female mice were severely sub-fertile but not completely infertile. Sub-fertility in these mice was defined as the ability to become pregnant but with a small delay in the onset of pregnancy [69,71,72]. It is noteworthy to mention that sub-fertility and infertility are two words with similar meanings, depending very much on the endpoints of the investigation and even more on the characteristics of the population. For example, the ovulation rate in mice is higher than in humans and a genetic deficiency that causes sub-fertility (longer time to achieve pregnancy) in mice might exhibit complete infertility in humans. It is crucial to define, especially in humans, subtle fertility phenotypes in order to overcome difficulties concerning human fertilization. Given that, with the exception of CD9, there are tetraspanins present on the oocyte’s surface creating an assembly of membrane microdomains, it is clear that these tetraspanins can not compensate for the deletion or the inactivation of CD9, which results in partial reproductive potential. Recently, the second large extracellular loop (LED) of the CD9 molecule and especially the amino-acid residues 173–175, has been suggested to play major role in the function of the CD9 molecule during spermatozoon–oocyte interaction [73]. It is likely that this region in the second loop of the CD9 interacts with other molecules of the oocyte’s surface making this area fusible [74].

Among the other tetraspanins CD81 is another well-characterized tetraspanin (in terms of function) with 45% identity to CD9. The reproductive performance of CD81 knockout mice is similar to that of CD9 knockout. Deletion of the CD81 coding region resulted in sub-fertility but the extent of this situation was relative milder than the sub-fertility observed in CD9-disrupted mice [75]. Interestingly, deletion of both CD9 and CD81 resulted in complete infertility, suggesting indirectly that these two surface molecules are interacting together, suggesting that the specific infertility is just a part of a more complicated situation [76]. This proposal seems to be reasonable if we take into account that during IVF the insemination of one oocyte by 80,000–100,000 prepared spermatozoa do not result always in fertilization success. Thus, fertilization failure may be explained by the failure of interaction between these two surface molecules. Nevertheless, as far as CD molecules are concerned, fertilization failure may also come from the side of sperm. Specifically, CD46 is a membrane cofactor protein (MCP), which is expressed only in acrosome-reacted spermatozoa [77] and is thought to be a factor that participates in the sperm–oocyte interaction, facilitating possibly the enzymatic cleavage of complement component C3b [78]. Although the exact mechanism has not been yet clarified it seems that CD46 binds to the C3b, which at low levels serves as a bridge between CD46 and CD11b/CD18 (α_M_β_2_), which is an integrin expressed solely on human oocytes [79]. The interaction between the CD46 of the spermatozoon and the α_M_β_2_ integrin of the oocytes is dependent on the concentration of the C3b. This means that saturating concentrations of the C3b molecule results in the inhibition of the interaction between spermatozoon and oocytes, but not necessarily causing infertility. The aberration of the CD46 coding region resulted in a new category of infertility, where the spermatozoa lost their capability to bind to the oocyte’s surface causing fertilization failure [80]. This proposal for fertilization failure also seems to be reasonable, given our experience in the IVF laboratory, where the insemination of one oocyte by 80,000–100,000 prepared spermatozoa do not result always in fertilization success. The prepared spermatozoa may perform poorly during fertilization because a large percentage of these spermatozoa may exhibit partial or complete disruption of CD46.

Although the above molecules have been studied in mice, in humans a different tetraspanin, CD151, has been described [81]. Mutated forms of CD151 appears not to have any negative reproductive result in mice, raising the possibility that different tetraspanin members perform variously in different mammalian species. With the exception of the CD molecules, integrins have been also described to participate in spermatozoon–oocyte adhesion. α6 integrin in combination with the β1 integrin subunit forms the VLA-6 (α6β1) integrin, which has been recognized on the surface of the oocytes [82]. Using α6β1 antibody tests, a 96% inhibition of the fusion process was found [81] and an association between α6β1 and CD9 at the oocyte surface was also shown [72]. The possible interaction between α6β1 and CD9 for the ADAM2 has not been yet clarified, but until now two models of fertilization have been considered. In the first one, ADAM2 of the acrosome-reacted spermatozoon binds directly to the α6β1 integrin of the oocyte’s surface, which is attached to the CD9 molecule and both molecules are in close proximity with the actin of the oocyte’s cytoskeleton. In the second model, ADAM2 binds again directly to the α6β1 integrin but this interaction is governed by the indirect communication between CD9, actin and α6β1 integrin [67]. Furthermore, α6 knockout oocytes showed normal fusibilty, suggesting that α6 integrin is not so essential for gamete membrane fusion [83]. It is possible that other surface integrins compensate the disrupted function of α6 integrin. Although data from knockout-integrin oocytes suggest that integrins are not responsible for infertility problems, it is noteworthy to state some oocyte integrins and their possible partners on the oocyte surface. It has been demonstrated that CD151 and CD81 co-regulate the function of α3β1 and α4β1 integrin, respectively, though the model of interaction between these two CD molecules and their respective integrins has not yet been clarified.

Among the players from the oocyte side, CD9 appears to be a key component of the multi-numeric CD molecules observed on the surface of the oocyte. Although the direct role of this molecule has not yet been elucidated, recent investigations recommended that CD9 knockout mice have a reduced ability to strongly adhere to spermatozoon [84], implying that the role of the CD9 molecule is placed in the binding phase of fertilization. This evidence shoots down the hypothesis that CD9 is the most essential molecule among the numerous molecules in the surface of the oocyte. Nevertheless, this suggestion was proposed a long time ago through experimental observations demonstrating CD9 knockout oocytes cannot be fused, but the spermatozoa were accumulated in the perivitelline space (the area between ZP and oocyte plasma membrane) [85]. It appears that CD9 does not have a direct role in the gamete fusion process, but it is very possible that CD9 accelerates or enhances sperm interaction or at last modifies the oocyte’s plasma membrane so as to be more fusogenic. Moreover, recent observations suggested that the CD9 molecule is secreted from the oocytes in vesicles, translocating to the sperm surface and promoting in this way the fusion process [86]. These vesicles are exosome-like structures and these CD9 exosomes seem to communicate with sperm in order to promote spermatozoon–oocyte membrane interactions [87,88]. This proposal appears to be very attractive since the exact molecular mechanism of the spermatozoon–oocyte membrane interaction has not been yet clarified. It is possible that these CD9-containing exosomes function as the chemoattractants released by the COCs to be “located” by the spermatozoa. Once the CD9 exosomes “open a dialog” with the molecules of the spermatozoon, the interaction is then completed by CD9 molecules on the oocyte’s surface. This raises the possibility that different regions of the CD9 molecule have different functions during the initiation and the completion of the fusion procedure.

Other candidate factors in the oocyte surface that are considered as potential fertilization proteins are the GPI-anchored proteins. These proteins are called rafts because they are enriched with lipid microdomains. Severe impairment of fusing ability when the GPI-anchored protein was disrupted was observed [89]. One reasonable explanation is that the absence of GPI-anchored proteins leads to the disruption of these lipid microdomains, which in turns alters the physiological composition of lipids on the oocyte’s surface, which inevitably results in the impairment of the interaction between spermatozoon and oocyte. Although an association between GPI-anchored proteins and tetraspanins has been proposed [90], this association has recently been indicated to be irrelevant [91] and it remains to be clarified whether these molecules interact for the fusion process.

Some candidates from the sperm side were proposed a long time ago for participating in the membrane fusion process. The epididymal protein, which is a cysteine-rich secretory protein, known as cysteine-rich secretory protein 1 (CRISP1), is one of the most well characterized sperm surface proteins. The identification of other related proteins result in the creation of the CRISP family. Besides the DE protein, which got its name by the presence of two bands, labeled the D and E bands on a denaturating polyacrylamide gel, another family member is cysteine-rich secretory protein 2 (CRISP2) CRISP2, which is mainly expressed in the testis [92,93] and cysteine-rich secretory protein 3 (CRISP3) with variable tissue distribution [94,95]. CRISP1 has been studied extensively in the rat and mouse. Experimental studies showed that CRISP1 knockout spermatozoa were incapable to fuse with the oocytes but able to bind to the oocyte’s surface [96,97]. CRISP1 is located in the dorsal region of the acrosome before acrosome reactions occur, but once acrosome exocytosis has taken place, CRISP1 migrates to the equatorial segment [98], the region where the spermatozoon fuses with the oocyte’s surface. In addition, it was found that CRISP1 could bind everywhere on the oocyte’s surface [99] except the region lacking microvilli (over the meiotic spindle), a site where it is known that spermatozoa cannot bind. This information allows us to speculate that there is a binding-partner molecule on the oocyte’s plasma membrane favoring the spermatozoon-oocyte membrane fusion. The human respective epididymal CRISP1 protein, known as the AEG-related protein (ARP), has been shown to have approximately 40% homology to rat CRISP1. Studies with anti-ARP antibodies concluded that the anti-ARP molecule reduces the fusion ability of the spermatozoa, but not the motility or binding ability [100].

Much attention has been drawn to the molecule IZUMO1, which is an approximately 56 KDa testis-specific member of the immunoglobulin superfamily (IgSF). It was first discovered as a monoclonal antibody that inhibits the fusion process [101]. Using two-dimensional gel electrophoresis followed by liquid chromatography-tandem mass spectrometry analysis, the antigen was named IZUMO, for a Japanese shrine dedicated to marriage. The gene encodes a transmembrane protein with an extracellular Ig domain, a single transmembrane region and a short cytoplasmatic tail [102]. IZUMO1 migrates from the anterior head of the spermatozoon to the equatorial segment during acrosome reaction, similarly to the migration of CRISP1 during acrosome reaction [102]. In addition, the fusion domain of the protein should be inactive in the equatorial segment before acrosome reaction takes place. The first indications that IZUMO1 was essential for the fusion process came from studies with IZUMO1 knockout mice. Specifically, IVF experimental assays with IZUMO1 knockout spermatozoa demonstrated that following penetration of the ZP, spermatovoa accumulate at the perivitelline space of the oocyte. Although intracytoplasmatic IZUMO1 knockout sperm injection (ICSI) resulted in normal embryos that implanted and developed, the exact role of IZUMO1 as a fusogen has not yet been clarified [102]. In addition, IZUMO1 does not contain any fusogenic peptide domain or domains similar to other fusion proteins reported in viruses [103]. Due to the lack of stereotypical features as a fusogenic, it is likely that the immunoglobulin-like domain interacts with other surface molecules [104]. For that reason much attention has been drawn to sperm proteins that interact with the immunoglobulin-like domain of IZUMO1, creating thus a fusogenic complex network. The identified IZUMO1-interacting protein was an ACE homologue, ACE3 and because it is expressed only in testis it was called tACE3. According to experimental data, knockout tACE spermatozoa demonstrated no signs of infertility [105], while immunofluorescent staining experiments showed that tACE3 is co-localized with IZUMO1 in the acrosomal cap before acrosome reaction, but once the acrosome reaction is performed, tACE3 disappears and IZUMO1 remains. Although IZUMO1 and tACE3 may cooperate to create fusogenic steering, experimental studies showed no complete absence of the reproductive performance [105]. Thus, investigations shifted to proteins that are located or expressed only in the equatorial segment of the sperm, known as sperm equatorial segment proteins (SPESPs). Among SPESPs, SPESP1 has received much attention because experiments with anti-SPESP1 antibodies resulted in severe inhibition of the spermatozoon-oocyte fusion in both hamsters and mice [106,107,108]. Furthermore, the deletion of SPEP1 resulted in the complete disruption of the sperm equatorial segment, while the MN antigen, which was found to participate in the fusion process, was located in the equatorial segment area after acrosome reaction. It has been proposed that SPESP1 might function in a way to restrain the MN antigen at the moment of fusion [109].

According to the above descriptions it is obvious that the membrane interaction procedure involves two distinct steps: The sperm binding where adhesion molecules are the main factors bringing membranes in a close apposition and the second step of membrane fusion. The second step is distinguishable from the first and involves the cytoplasmatic continuity between spermatozoon and oocyte. Two models have been proposed for the gamete plasma membrane interaction and a schematic presentation of one of the hypothetical models was demonstrated by Evans [110]: Briefly, on the sperm surface there is a fusion protein that contains a folded hydrophobic fusion domain. Before fusion occurs, the adhesion procedure should take place in order that the two plasma membranes come into close apposition. Adhesion is mediated by two surface proteins, which function as a receptor—Ligand pair in each plasma membrane. One of the pair proteins, the one that is closer to the site where the fusion opening will be made, makes a bend in the lipid bilayers so the two membranes come in contact. At that time the fusion protein undergoes a structural change exposing the fusion domain and inserting it in the opposite membrane bilayer. Next, the outer leaflets of the two bilayers intermingle (hemi-fusion) and create an opening known as the fusion pore. The incorporation of one membrane into the other results in the connection between the cytoplasms of the two gametes [110]. Although the model is very attractive, it is not known whether adhesions–hemi-fusions–fusions are steps which follow the “switch on or switch off” model. In other words, we do not know whether adhesion or hemi-fusion will result in the creation of the fusion pore, accomplishing in this way the fusion procedure. How important the fusion domain is, and whether the fusion process would proceed if this domain undergoes the structural change as described above, is not clear. Also, what are the conditions that adhesion and hemi-fusion are promoted? Although we know much about the fusion processes of viruses, which in some way are similar to the fusion process of fertilization, further study on these issues will shed more light in the so-called fusion process of fertilization. According to the above model, in addition to the molecules involving in the adhesion/fusion process we can assume that one of the pairs of proteins described above may be CD9 in combination with α6β1 integrin from the oocyte side, and IZUMO1 in combination with SPESP1 from the spermatozoon side.

In contrast to the almost established knowledge that the CD9 oocyte molecule is the partner of the IZUMO1 sperm molecule during fusion, recent evidence supports the evolutionary notion that the partner of IZUMO1 in the oocyte side is the folate receptor 4 (Folr4), named Juno for the Roman goddess of fertility and marriage [111]. In this study, after a number of sequential IVF experiments, it was proposed that the oocytes of knockout Juno mice were completely incapable to be fertilized by acrosome-reacted spermatozoa, while the percentage of fertilization followed in a Juno concentration-dependent manner [111], meaning that low dispersal of Juno receptors in the oocyte surface reduces the fertilization potential, while complete absence of Juno receptors in the oocyte surface leads to complete fertilization failure. The same study also proposed that due to the presence of the tetraspanin web on the oocyte surface, CD9 is the molecule that helps the Juno receptor to interact with sperm IZUMO1. This suggestion is also very possible since it was so far understood that CD9 helps other unknown oocyte surface molecules to interact with IZUMO1. The discovery of Juno seems to be a breakthrough in the fertilization process, since IZUMO1 finds finally his mate [112]. Furthermore, in the previous study, it was well established that Juno-deficient mice were completely infertile in contrast to CD9-deficient mice, which were only partially infertile. It is very possible that total fertilization failure depends very much upon the distribution of Juno receptors in the oocyte surface rather than the fertilization ability of the spermatozoa. Nevertheless, the absence of a fusogenic domain in the Juno molecule promotes the notion that the specific molecule is essential for the adhesion part of the fertilization procedure. It is very possible that the interaction of IZUMO1-Juno is essential for the promotion of the last part of the fertilization, which is the fusion process. Moreover, it also very possible that Juno dispersion is proportional to microvilli dispersion, since the processes of adhesion and fusion take place only if microvilli are abundant. Recently, it was proposed that syncytin-1, acting as fusogenic on the sperm side, and ASCT-2 acting as a receptor on the oocyte side are some of the many cellular molecules that finally participate in the fusion process of fertilization [113], but need to be clarified with additional IVF experimental studies. Nevertheless, the need to elucidate the molecular mechanisms of fertilization is a matter of human IVF experimental studies, something which is rather difficult to overcome due to the nature of the studies, but the use of the embryoscope is a good choice to clarify the exact mechanism as commented by Albertini [114].

## 3. Fertilization in the IVF Era-Conclusion Remarks

It is obvious that our understanding regarding the molecular and cellular phases of fertilization is rapidly increasing due to advances in assisted reproductive technologies. Human *in vitro* fertilization (IVF) and intracytoplasmatic sperm injection (ICSI) have been developed recently (30 years) and are developing continuously to overcome male and female infertility problems. The understanding of the underlying mechanisms that governs fertilization may help clinicians to provide exact information about the infertility problems that couples encounter regarding fertilization or even embryo development and implantation. It is known that during an IVF/ICSI treatment the semen sample is being prepared by two methods: the swim-up, and the Percoll. Both methods obtain acrosome-reacted spermatozoa; this means that the first steps of fertilization, including migration, are bypassed. Whether the molecules that participate in the migration process abide to the sperm surface after sperm preparation is not known. Nevertheless, the factors that inhibit the acrosome reaction are discarded during sperm preparation. According to the descriptions of molecules that participate in the general fertilization process, the ADAM family, fertilin α, fertilin β and cyritestin, are the most likely candidate factors involved in the migration process. Furthermore, ADAM2 seems to be intermediate in the adhesion process during the fusion between the plasma membranes. For that reason it is essential to realize if some sperm surface molecules are discarded during sperm preparation, because the removal of such molecules may produce spermatozoa ready for acrosome reaction but not potent to fertilize. This is clearly one problem that clinical embryologists encounter when they prepare a semen sample for conventional IVF insemination. Specifically, it is common knowledge that every cumulus oocyte complex (COC) needs about 80,000–100,000 ready for acrosome reaction of straight forward motion spermatozoa in order to obtain a normal fertilization rate of approximately 65%–75%. The remaining 25%–35% seems to be attributed mainly to the immaturity of the oocytes and secondly to a variety of known factors amongst semen sample and culture media. If we take into consideration that during semen preparation a large percentage of sperm surface molecules become incapable to function or are detached from the sperm, then the 80,000–100,000 prepared spermatozoa are not enough to accomplish a 100% fertilization rate, because only a portion of the above acrosome-reacted spermatozoa are potent fertilizers. The above suggestion is very likely to happen because during observations for the presence of two pronuclei the next day (normal fertilization) many spermatozoa are found on the ZP of the oocytes which failed to be fertilized (no formation of pronuclei), meaning that some acrosome-reacted spermatozoa cannot penetrate the ZP or cannot interact with the plasma membrane of the oocytes in order for fusion to occur. It should be noted that spermatozoa that bind to the ZP are not all acrosome-reacted, and that not all acrosome-reacted spermatozoa can penetrate the ZP. This may imply that there are perhaps different populations in the same semen sample that can explain the above situation. Moreover, the different populations in the same semen sample can be otherwise explained by evolutionary theory, where one spermatozoon will succeed in fertilization among the millions in one semen sample. Secondly, we should also bear in mind that the number of spermatozoa used for insemination is according to the straight-forward motility status without the embryologists having the ability to check the acrosome status. It is hypothetically believed that the 80,000–100,000 spermatozoa per COC correspond to 80,000–100,000 acrosome-reacted spermatozoa. This is therefore one explanation for relatively low fertilization rates in the IVF treatments. Another explanation is the absence of molecules on the oocyte surface. It is well established that ovarian stimulation effects the quality of the oocytes and this impact may be extended to the molecules on the oocyte plasma membrane or even to the microvilli. It is very possible that the impairment of the microvilli in combination with the destruction of molecules, such CD9, that participate in the general sperm-oocyte interaction leads inevitably to the inability of sperm surface molecules to bind the molecules of the oocyte surface.

The breakthrough of the identification of the IZUMO1 mate [111] provides novel insights for the rational development of new fertility treatments. Specifically, in the same study during IVF assays it was demonstrated that 0.1 µg·mL^−1^ or higher concentrations of anti-Juno monoclonal antibodies potently prevented fertilization. The Juno concentration-dependent fertilization potential of the oocytes, as indirectly supported by the last study, directly supports the notion that fertilization failure is a matter of molecular mechanisms based mainly on receptors and their affinity to their binding partners. Furthermore, from the IVF point of view, this notion is very tempting if we take into account that during conventional IVF almost all acrosome-reacted spermatozoa are carrying the IZUMO1 molecule, and fertilization success depends on the concentration-dependent dispersion of Juno. Due to the fact that the average percent of fertilization success during the conventional IVF is approximately 75%, it is very possible that lower or zero fertilization percentages may be attributed to the lower or complete absence of Juno receptors in the oocyte surface. Given that conventional IVF is correlated to excellent sperm parameters, it is very possible that fertilization failure may be attributed to cellular and molecular mechanisms involving the Juno receptors, and that as the concentrations of Juno on the oocyte surface increase, the fertilization percentages proportionally increase as well. Although the above considerations are very attractive and likely very close to reality, further molecular studies in combination with IVF studies are needed to establish the molecular mechanisms of fertilization.

Furthermore, many factors/molecules present in the follicular or oviduct fluid favor the positive interaction between COCs and ovulated oocytes in the female reproductive tract, they cease to exist soon after ovum collection. For example, the molecule C3b (mentioned above) which functions in a concentration-dependent manner for the interaction between CD46 of the spermatozoon and the α_M_β_2_ integrin of the oocytes, is not present after oocyte collection, preventing the possible interaction between sperm CD46 and oocyte α_M_β_2_ integrin. Nevertheless, fertilization occurs, demonstrating that these proteins are not essential for the whole fertilization process.

Except for the mechanisms involved in the oocyte-spermatozoa interactions, there are also several factors that may affect the fertilization potential of the spermatozoa. The most known is the presence of the human papilloma virus (HPV), which has been described to persist even after cryopreservation [115]. Most results are coming from IVF clinical studies. Although the mechanism of infection of HPV in spermatozoa is not yet clarified, it is known that HPV is located at the equatorial region of the sperm head (the region that participates in the oocyte-spermatozoon fusion) through interactions between the HPV capsid protein L1 and syndecan-1, functioning in this way as a vector for HPV transfer into the oocytes [116,117]. During the sperm preparation procedure in IVF-ET (embryo transfer) treatments, it has been suggested that semen washing rarely eliminates HPV sperm infection at least in infertile patients [118], while the treatment with Heparinase-III seems to reduce the risk of HPV infection when using assisted reproduction techniques [119], something that needs to be clarified by further studies. A 24-month follow-up is needed to obtain a significant clearance of both HPV and anti-sperm antibodies for candidate-patients of assisted reproduction [120]. Overall, HPV has been demonstrated to affect sperm characteristics, without nevertheless affecting the fertilization ability of the spermatozoa [121]. More studies are needed to elucidate the molecular impact of HPV on the mechanism of oocyte–spermatozoon interaction.

**Figure 4:**
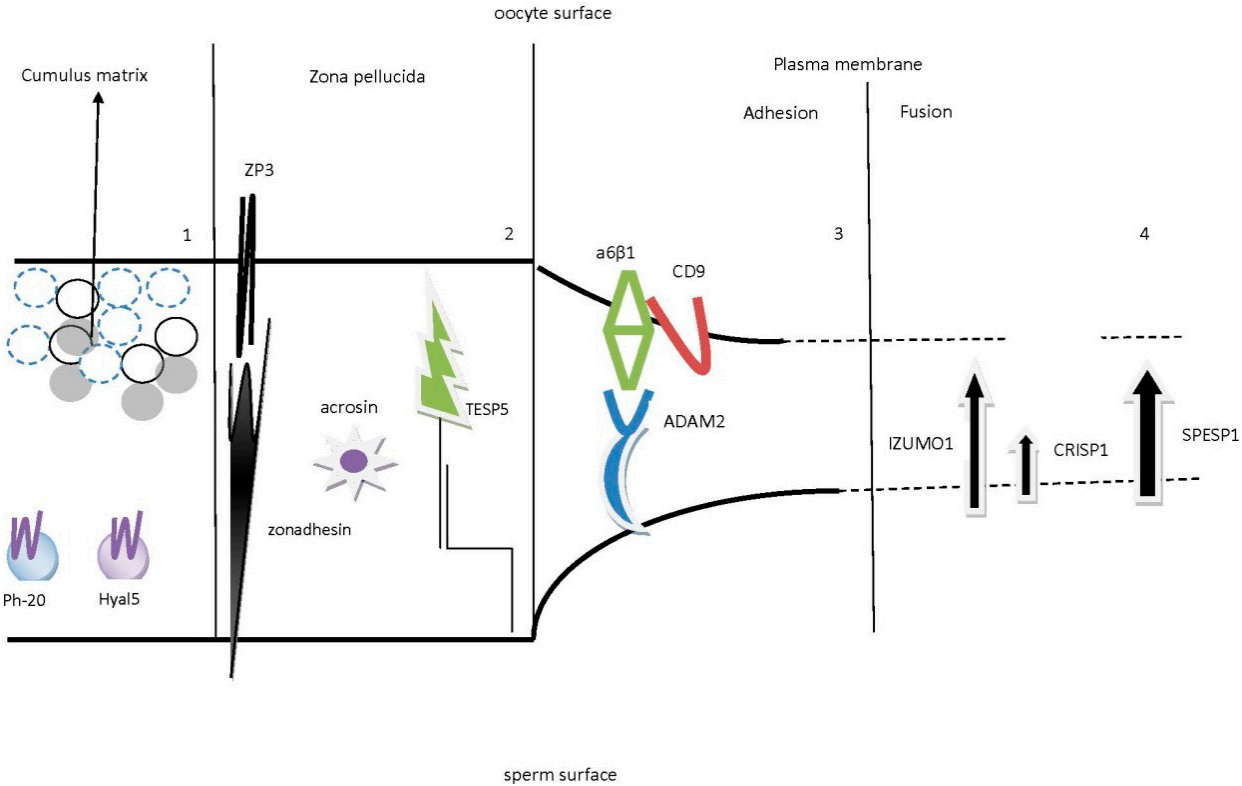
A hypothetical model of all the molecules that participate in the multi-step process of fertilization, initiating from the penetration of the cumulus matrix, proceeding with the attachment, binding and penetration of the ZP and ending with the adhesion and fusion of the two gamete plasma membranes. Most molecules seem to have a partner on the other side, forming a pair, while several molecules, especially from the sperm side, function as trypsin-like molecules. In the first compartment, the cumulus matrix is believed to be dispersed by the molecule Hyal5 and the cumulus cells are then digested by the enzyme Ph-20. We should note that in this phase the acrosome reaction has not yet initiated because these molecules are located in acrosome-intact spermatozoa. The second compartment displays the interaction between spermatozoon and the ZP. In this phase of adhesion, zonadhesin (and sp56) from the sperm side has been suggested to interact with a specific domain of the O-linked oligosaccharide of the ZP3, known as the combining-site. In the phase of ZP penetration, acrosin is the molecule that is released during acrosome reaction, while TESP5, a spermatozoon GPI-anchored membrane serine protease, has been proposed to co-operate with acrosin until ZP penetration is accomplished. In the third compartment (where the plasma membranes are in close apposition) the molecules that participate in the adhesion of spermatozoon to the plasma membrane of the oocyte are depicted. Experimental studies revealed that the CD9 molecule, forming a “tetraspanin web”, cooperates with α6β1 integrin in the oocyte plasma membrane in order to interact with ADAM2 of the sperm surface. Finally, the two gamete membranes are approaching, a situation which is known as hemi-fusion. The fourth compartment describes the molecules participating in the last phase of interaction, known as the fusion process. The fusion pore is obtained by the function of IZUMO1 of the sperm surface, while the CRISP1 molecule has been recognized to assist in the fusion process. Recently the Juno molecule, a folate receptor 4 (Folr4), has been proposed as the partner of IZUMO1. IZUMO1 and Juno function as a pair for the adhesion part of the fertilization process, and this interaction seems to be essential for the final step of fertilization, the fusion process. Due to the fact that the equatorial segment of the spermatozoon is the direct site that comes in close apposition with the oocyte surface, SPESP1 has been implicated in the formation of the fusion pore.

Ovarian stimulation on the one hand and semen preparation on the other seem to negatively impact on molecules that play key roles in the general sperm–oocyte interactions (Figure 4). The understanding so far in conventional IVF insemination that the relative low fertilization rate is attributed to the oocyte quality and to the culture environment seems to be gradually questioned, while the destruction of critical molecular interaction hypothesis appears to gain more ground. Experimental IVF studies should be performed in order to clarify the molecular basis of infertility in regards to fertilization.
